# Evaluation of predictive factors for i-CLARAS (intraoperative complications in laparoscopic renal and adrenal surgery): a multicentre international retrospective cohort study

**DOI:** 10.1038/s41598-024-51696-2

**Published:** 2024-01-16

**Authors:** Angelo Territo, Giuseppe Di Buono, Salvatore Buscemi, Guglielmo Mantica, Vincenzo Falco, Vital Hevia Palacios, Paolo Verri, Rodrigo Antelo Antelo, Jesus Emmanuel Rosas-Nava, Nicolae Crisan, Iulia Andras, Fabio Medas, Giuseppe Amato, Giorgio Romano, Alberto Breda, Antonino Agrusa, i-CLARAS (intraoperative Complication in Laparoscopic Renal and Adrenal Surgery) Research Collaborative Study Group, Ferdinando Agresta, Nicoletta Adelfio, Danilo Amparore, Gabriele Anania, Giuseppe Badalamenti, Francesco Bagolini, Gabriele Barletta, Umberto Bracale, Maximilian Buzoianu, Pietro Giorgio Calò, Gian Luigi Canu, Giuseppe Cicero, Roberto Citarrella, Pietro Coletta, Francesco Corcione, Diego Cuccurullo, Gaspare Cucinella, Francesco Cupido, Francesco D’Angelo, Carlo Feo, Ana Domínguez Gutiérrez, Andrea Gallioli, Jose Maria Gaya, Girolamo Geraci, Gerardo Tena Gonzales-Mendez, Mario Guerrieri, Giuseppe Gullo, Bianca Iacone, Isaac Roberto Labra Salgado, Edelweiss Giulia Licitra, David Lopez Curtis, José Antonio López Plaza, Matilde Micheli, Giulia Montori, Nadav Nevo, Dario Oppici, Leandro Arellano, Monica Ortenzi, Miriam Palmieri, Antonio Piccione, Francesco Porpiglia, Pablo Raffaele, Stefano Reggio, Giorgio Romano, Gaia Russo, Raul Sanchez-Molina, Isabel Sanz Gomez, Marta Saverino, Maria Grazia Sibillla, Gianfranco Silecchia, Antonio Stigliano, Anna Tedesco, Teodora Telecan, Carlo Terrone, Maria Rosaria Valerio, Francesco Vecco, Roberta Vella, Francesco Vitale

**Affiliations:** 1 Department of Urology, Fundació Puigvert, Autonoma University of Barcelona, Barcelona, Spain; 2 Department of Surgical, Oncological and Oral Sciences, University of Palermo, Via L. Giuffrè, 5, 90127 Palermo, Italy; 3 Department of Surgical and Diagnostic Integrated Sciences (DISC), University of Genova, Genova, Italy; 4 Department of Economics, Business and Statistics, University of Palermo, Palermo, Italy; 5 Urology Department, Hospital Universitario Ramón y Cajal, Alcalá University, Madrid, Spain; 6 Division of Urology, Department of Oncology, University of Turin, San Luigi Gonzaga Hospital, Turin, Italy; 7 Unidad Renal Fundacion Favaloro, Buenos Aires, Argentina; 8 Hospital General de Mexico, Universidad Nacional Autonoma de Mexico, Mexico City, Mexico; 9 Urology Department, Clinical Municipal Hospital Cluj-Napoca, Cluj-Napoca, Romania; 10 Department of Surgical Sciences, University of Cagliari, Cagliari, Italy; 11 Department of Surgery, Vittorio Veneto General Hospital, Vittorio Veneto, Italy; 12 Department of Morphology, Surgery and Experimental Medicine, University of Ferrara, Ferrara, Italy; 13 Department of Medicine, Surgery and Dentistry, University of Salerno, 84084 Salerno, Italy; 14 Department of General Surgery, Clinica Mediterranea, 80122 Naples, Italy; 15 Department of General, Laparoscopic, and Robotic Surgery, Ospedale Monaldi, Azienda Ospedaliera dei Colli, Naples, Italy; 16 Department of Obstetrics and Gynecology, Villa Sofia Cervello Hospital, IVF UNIT, University of Palermo, 90146 Palermo, Italy; 17 Department of Medical and Surgical Sciences and Translational Medicine, Faculty of Medicine and Psychology, St Andrea Hospital, Sapienza University, Rome, Italy; 18 Department of General and Emergency Surgery, Marche Polytechnic University, Ancona, Italy; 19 Servicio de Urología, Hospital General de México “Dr. Eduardo Liceaga”, Mexico City, Mexico

**Keywords:** Urological cancer, Endocrinology, Urology, Endocrine system and metabolic diseases, Kidney diseases

## Abstract

The laparoscopic approach represents the standard of treatment for renal and adrenal diseases, and its use is increasing even outside referral centres. Although most procedures are routinely performed, intraoperative complications do not occur, and the rate and predictive factors of these complications have not been established. The aim of this study was to evaluate the incidence and type of intraoperative complications and to identify predictive factors in patients undergoing laparoscopic renal and adrenal surgery. This was a cohort, multicentre, international retrospective study. Patients who underwent laparoscopic renal and adrenal surgeries between April 2017 and March 2022 were included in the study. Bivariate analysis was performed using contingency tables and the χ^2^ test for independent samples to compare qualitative variables and the T test and Mood test for continuous variables. Multivariate analysis was performed using a logistic regression model to obtain adjusted odds ratios. A total of 2374 patients were included in the study. Intraoperative complications were reported for 8.09% of patients who underwent renal surgery, with the most common complications reported being hollow viscus and vascular complications, and for 6.75% of patients who underwent adrenal surgery, with the most common complication reported being parenchymatous viscous complications. Multivariate analysis revealed that both adrenal and renal surgery radiological preoperative factors, such as invasive features during adrenalectomy and the RENAL score during nephrectomy, are predictive factors of intraoperative complications. In contrast to existing data, surgeon experience was not associated with a reduction in the incidence of perioperative complications.

## Introduction

Since the use of the laparoscopic approach for adrenalectomy was initially reported by Gagner et al. in 1992^1^, laparoscopic adrenalectomy (LA) has been the standard of care for the treatment of all benign adrenal masses because it is associated with reduced postoperative pain, early oral intake, and short hospital stays^2^. During the same period, Clayman published the first case series on laparoscopic nephrectomy, reporting the same advantages as the minimally invasive approach^3^. Since then, the use of laparoscopy, including partial nephrectomy (LPN) and nephroureterectomy for urothelial upper tract carcinoma, for accessing retroperitoneal organs has increased rapidly.

Furthermore, with improvements in technology and the use of new surgical techniques, such as the retroperitoneal approach, laparoscopic surgery has been meaningfully improved and increasingly adopted to the extent that the laparoscopic approach to renal surgery, for both radical and partial nephrectomy, is considered the standard procedure at many institutions whenever feasible. Compared with open surgery, laparoscopic surgery has been proven to have identical long-term oncologic outcomes^4–7^ and added benefits, such as shorter hospital stays, lower analgesic requirements^8,9^, and shorter convalescence times. Therefore, despite the widespread use of robotic surgery, the purely laparoscopic approach is still considered the treatment of choice for many benign and malignant diseases, including complex cases for which surgery^10^, such as general and endocrine surgery, as well as urologic surgery (0.7–5.4%) may be difficult. Nevertheless, potentially life-threatening complications during laparoscopic renal and adrenal surgery, including bowel injury (0.8%)^11^, spleen injury (1.4%), pancreatic injury (0.4%)^12^, diaphragmatic injury (0.6%)^13,14^, and vascular complications (0.7–5.4%) are still being reported^15^. Indeed, accessing the retroperitoneal space is a challenge for laparoscopic surgeons because of the need to carefully control veins and arteries that are located deep, behind other structures, and in close proximity to the hollow and parenchymatous viscus. The aim of this study is to determine the rate of intraoperative complications of adrenal and renal surgery by retrospectively examining a large international multicentre database and to identify the predictive factors of perioperative complications.

## Methods

**Figure 1:**
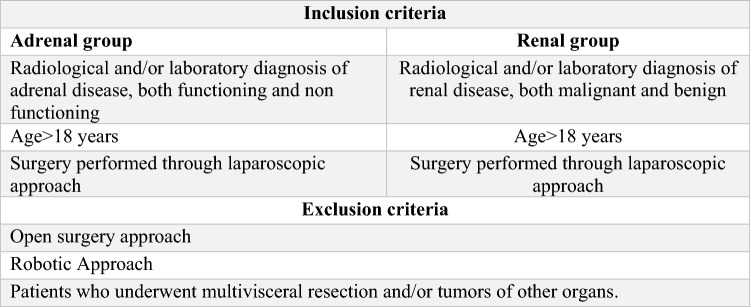
Inclusion and exclusion criteria.

This multicentre international retrospective study included patients who underwent laparoscopic renal and adrenal surgery between April 2017 and March 2022. Seven centres were in Italy (Palermo, Roma, Ancona, Napoli, Torino, Genova, Cagliari), two were in Spain (Madrid, Barcelona), one was in Mexico, one was in Argentina, and one was in Romania (i-CLARAS Study Collaborative Group). Only patients treated by the laparoscopic approach were considered because not all participating centres have access to the robotic platform. This clinical study is referred to as the i-CLARAS (intraoperative Complication in Laparoscopic Renal and Adrenal Surgery) study and was publicly registered and approved by the ethics committee of the promoting centre (University Hospital Policlinico of Palermo). All the research was performed in accordance with the relevant guidelines. Informed consent was obtained from all participants and/or their legal guardians. This work has been reported in line with the STROCSS criteria^16^. The inclusion and exclusion criteria are presented in Fig. 1.

*Preoperative data*. The following preoperative data were retrospectively collected: age, BMI, sex, comorbidities (hypertension and diabetes), and preoperative estimated glomerular filtration rate (GFR). To evaluate patients’ general performance status, the Charlson Comorbidity Index was calculated, and the ASA score was collected for each patient. The preoperative bleeding risk was assessed, and the following aetiologies were recorded: previous surgery, direct-acting oral anticoagulants (DAOCs), or haematologic disease. In the renal group, details on the preoperative diagnosis were collected, including the pathological characteristics (benign or malignant disease) and details regarding the pathological diagnosis. Malignant diseases were classified as clear cell renal cell carcinoma (ccRCC), papillary renal cell carcinoma (RCC), chromophobe RCC, Bellini RCC, unclassified, or other. Benign diseases were classified as oncoytoma, renal angiomyolipoma (AML), xanthogranulomatous pyelonephritis, or polycystic kidney/renal cyst. For malignant diseases, the preoperative extent of the primary tumour according to the TNM staging system was previously reported. The following preoperative radiological data were collected: side (right or left), PADUA score, PADUA risk category score, and RENAL score. In the adrenal group, the following preoperative data were collected for each patient: lesion side, number of lesions, greatest dimension (mm), presence of invasive features and/or organ invasion, and details of the pathology (functioning, nonfunctioning, or malignant disease). The following operative data were collected: operative time; surgeon experience (young or senior surgeon with a cut-off of 30 procedures); surgical approach (transperitoneal, retroperitoneal, or hand-assisted); type of intervention (for the renal group: partial nephrectomy, radical nephrectomy, nephroureterectomy, pyeloplasty, or pyelolithotomy; for the adrenal group: adrenalectomy and adrenal sparing surgery); intraoperative blood transfusion; and drain use. In both groups, the following operative data were collected regarding intraoperative complications: the occurrence of intraoperative complications; the cause of damage (trocar placement, surgical manoeuvre, instrument malfunction, other); the type of vascular complications (minor: adrenal vessels, accessory adrenal vessels or other; major: renal artery, renal vein, vena cava, suprahepatic veins, or other); the resolution of vascular complications; the type of parenchymatous viscous complication; the resolution of parenchymatous viscous complications; the presence of hollow viscus complications; and the resolution of hollow viscous complications. The following postoperative data were collected: length of hospitalization (days), unplanned intensive care unit (ICU) admission, postoperative blood transfusion, short-term postoperative complications and 30-day postoperative complications. All postoperative complications were classified according to the Clavien–Dindo classification.

### Statistical analysis

All the data are presented as the mean ± standard deviation (SD) for continuous variables, median (interquartile range) for ordinal variables, and contingency tables for qualitative variables. Univariate and multivariate analyses were performed to assess the association between preoperative and intraoperative data and the rate of intraoperative complications. Bivariate analysis was performed using the chi-square test for independent samples for qualitative variables, and the t test and Mood test were used for continuous and ordinal variables, respectively. Multivariate analysis was performed, and odds ratios (ORs) with 95% confidence intervals (CIs) were calculated via logistic regression models. A two-tailed, *P* value < 0.05 indicated statistical significance. Statistical analysis was conducted by a biomedical statistician using R software (Core Team, Vienna, Austria, 2013).

### Ethics approval and consent to participate

The study was approved by the Ethical Committee “Comitato Etico Palermo 1” (No. 06/2022–14/06/2022) of the Policlinic of the University of Palermo and registered on clinicaltrials. gov (NCT05322265).

## Results

We performed a retrospective study, and we selected patients on the basis of the inclusion criteria. On the basis of this selection, a total of 2374 patients who underwent laparoscopic renal and adrenal surgeries were included in the study. Moreover, we excluded 12 patients from the study for whom the type of complication and/or treatment performed was unclear. Preoperative data are summarized in Table 1.

**Table 1:** Preoperative, intra-operative and post-operative characteristics of patients in the adrenal surgery (*n* = 409) and Preoperative, intra-operative and post-operative characteristics of patients in the renal group (n = 1965).

Preoperative, intra-operative and post-operative characteristics of patients in the adrenal surgery (*n* = 409)			Preoperative, intra-operative and post-operative characteristics of patients in the renal group (n = 1965)		
Pre-operative variables	Age	55.17 ± 13.80 (range 18–86)	Pre-operative variables	Age	59.51 ± 14.87 (range 18–81)
	Gender			Gender	
	Male	172 (42.05)		Male	1057 (53.79)
	Female	237 (57.95)		Female	900 (45.80)
	BMI	27.63 ± 5.61 (range 15.8–43)		BMI	26.89 ± 4.84 (range 18–60)
	Diabetes			Diabetes	
	Type 1	15 (3.67)		Type 1	54 (2.75)
	Type 2	63 (15.40)		Type 2	171 (8.70)
	ASA	2 (2.00–3.00)		ASA	2.00 (1.00–3.00)
	CCI (Charlson’s Comorbidity Index)	2.00 (1.00–4.00)		CCI (Charlson’s Comorbidity Index)	3.00 (2.00–4.00)
	Increased preoperative bleeding risk	93 (28.79)		Increased preoperative bleeding risk	586 (29.82)
	Pathological characteristics			Pathological characteristics	
	Functioning adenoma	184 (44.99)		Malignant disease	1339 (68.53)
	Non functioning adenoma	157 (38.39)		Benign disease	615 (31.47)
	Malignant	68 (16.62)		Median PADUA score	7.00 (6.00–8.00)
	Median tumor size (mm)	46.00 (30.00–65.00)		Median RENAL score	6.00 (5.00–7.00)
Intra-operative variables	Operative time (min)	125.00 (80.50–180.00)	Intra-operative variables	Operative time (min)	150.00 (110.00–200.00)
	Surgeon experience			Surgeon experience	
	Senior surgeon	390 (95.35)		Senior surgeon	1813 (92.26)
	Young surgeon	18 (4.41)		Young surgeon	145 (7.38)
	Type of intervention			Type of intervention	
	Adrenalectomy	406 (99.27)		Partial nephrectomy	849 (43.21)
	Adrenal sparing surgery	2 (0.49)		Radical nephrectomy nephroureterectomy	827 (42.09) 120 (6.11)
				Pyeloplasty	128 (6.51)
				Pilolithotomy	34 (1.73)
	Surgical approach			Surgical approach	
	Transperitoneal	382 (93.40)		Transperitoneal	223 (52.5)
	Retroperitoneal	26 (6.36)		Retroperitoneal	200 (47.1)
	Intra-operative complications	27 (6.75)		Intra-operative complications	123 (6.26)
	*Vascular complications*	4 (14.81)		*Vascular complications*	43 (39.3)
	*Hollow viscus complications*	2 (7.41)		*Hollow viscus complications*	49 (39.84)
	*Parenchymatous viscus complications*	13 (48.15)		*Calyx damage*	28
				*Renal pelvis lesion*	7
				*Ureter lesion*	2
				*Vagina lesion*	1
				*Total Bowel lesion*	10
				*Ileum lesion*	4
				*Colon lesion*	6
				*Parenchymatous viscus complications*	31(25.20)
	Conversion rate	14 (3.42)		Conversion rate	16 (0.81)
	*Among patients who experienced intraoperative complications*	13 (48.15)		*Among patients who experienced intraoperative complications*	16 (13.01)
	Drainage	256 (62.59)		Drainage	1567 (79.74)
	Intraoperative blood transfusion	2 (0.49)		Intraoperative blood transfusion	19 (0.97)
Post-operative variables	Short term complications	55 (13.45)	Post-operative variables	Short term complications	323 (16.44)
	30 days postoperative complications	2 (0.49)		30 days postoperative complications	165 (8.40)
	Postoperative blood transfusion	9 (2.20)		Postoperative blood transfusion	92 (4.68)

### Intraoperative complications

Intraoperative complications were reported for 8.09% (n. 123) of patients who underwent renal surgery and for 6.75% (n. 27) of patients who underwent adrenal surgery. In the adrenal group, the most frequent complications reported were parenchymatous viscous complications (48.15%), followed by vascular complications (14.81%) and hollow viscus complications (7.41%) (Tables 1 and 2). Particularly, for patients who underwent adrenal surgery and who had invasive features, the rate of intraoperative complications was 25%, and the most frequent complications were parenchymatous viscous complications (3 patients, one patient had splenic injury, and two patients had other parenchymatous viscous lesions); one patient also experienced vascular complications, and no patient experienced hollow viscus complications. In the renal group, the most frequent complications were hollow viscus (39.84%) and vascular (39.3%), followed by parenchymatous viscous complications (25.20%).

**Table 2:** Univariate analysis for factors associated with intraoperative and short term post-operative complications in the adrenal surgery group (*n* = 409).

	Intraoperative complications		*p*-value		Short term complications		*p*-value
	0 (*n* = 373)	1 (*n* = 27)			0 (*n* = 265)	1 (*n* = 55)	
Age (NA = 26)	55.29 ± 13.74	52.61 ± 14.05	0.357	Age (NA = 26)	55.35 ± 13.69	54.89 ± 14.78	0.832
Gender				Gender			
Male	155 (91.2)	15 (8.8)	0.223	Male	104 (75.4)	34 (24.6)	**0.003**
Female	218 (94.8)	12 (5.2)		Female	161 (88.5)	21 (11.5)	
BMI (NA = 204)	27.61 ± 5.76	28.89 ± 5.02	0.322	BMI (NA = 204)	27.71 ± 5.86	26.51 ± 4.09	0.340
Diabetes (NA = 90)				Diabetes (NA = 90)			
Type 1	15 (100.0)	0 (0.0)	0.173	Type 1	11 (100.0)	0 (0.0)	0.196
Type 2	55 (87.3)	8 (12.7)		Type 2	47 (77.0)	14 (23.0)	
ASA (NA = 42)	2.00 (2.00–3.00)	3.00 (2.00–3.00)	0.090	ASA (NA = 42)	2.00 (2.00–3.00)	3.00 (2.00–3.00)	< 0.001
CCI (NA = 152)	2.00 (1.00–4.00)	2.00 (2.00–3.25)	0.738	CCI (NA = 152)	2.00 (1.00–3.00)	3.00 (2.00–5.25)	**0.013**
Tumor size (NA = 3)	47.50 (30.00–65.00)	45.00 (26.00–60.00)	0.578	Tumor size (NA = 3)	45.00 (30.00–60.00)	51.00 (31.00–70.00)	0.513
Invasive features (NA = 151)	9 (75.0)	3 (25.0)	0.089	Invasive features (NA = 151)	7 (63.6)	4 (36.4)	0.254
Increased preoperative bleeding risk (NA = 86)	85 (91.4)	8 (8.6)	0.818	Increased preoperative bleeding risk (NA = 86)	57 (69.5)	25 (30.5)	**0.001**
Pathology				Pathology			
Functioning	167 (93.3)	12 (6.7)	0.322	Functioning	127 (83.0)	26 (17.0)	0.498
Non functioning	140 (91.5)	13 (8.5)		Non functioning	100 (84.7)	18 (15.3)	
Malignant	66 (97.1)	2 (2.9)		Malignant	38 (77.6)	11 (22.4)	
Operative time (NA = 2)	120.00 (80.00–176.25)	159.00 (130.00 –243.00)	**0.016**	Operative time (NA = 2)	120.00 (75.00–160.00)	175.00 (132.50–242.50)	**< 0.001**
				Intraoperative complications (NA = 9)			
				0	250 (84.7)	45 (15.3)	**0.010**
				1	15 (62.5)	9 (37.5)	
Surgeon (NA = 1)				Surgeon (NA = 1)			
Senior surgeon	358 (93.7)	24 (6.3)	0.113	Senior surgeon	259 (82.7)	54 (17.3)	0.999
Young surgeon	15 (83.3)	3 (16.7)		Young Surgeon	6 (85.7)	1 (14.3)	
Type of intervention (NA = 1)				Type of intervention (NA = 1)			
Adrenalectomy	371 (93.2)	27 (6.8)	0.999	Adrenalectomy	263 (82.7)	55 (17.3)	0.999
Adrenal sparing surgery	2 (100.0)	0 (0.0)		Adrenal sparing surgery	2 (100.0)	0 (0.0)	
Surgical approach (NA = 1)				Surgical approach (NA = 1)			
Transperitoneal	353 (93.1)	26 (6.9)	0.999	Transperitoneal	244 (81.9)	54 (18.1)	0.143
Retroperitoneal	20 (95.2)	1 (4.8)		Retroperitoneal	21 (95.5)	1 (4.5)	
Drainage (NA = 23)				Drainage (NA = 23)			
No	129 (99.2)	1 (0.8)	**< 0.001**	No	84 (86.6)	13 (13.4)	0.331
Yes	230 (89.8)	26 (10.2)		Yes	181 (81.9)	40 (18.1)	
Intraoperative blood transfusion (NA = 179)				Intraoperative blood transfusion (NA = 179)			
No	210 (92.1)	18 (7.9)	**0.007**	No	180 (93.3)	13 (6.7)	0.139
Yes	0 (0.0)	200 (100.0)		Yes	1 (50.0)	1 (50.0)	
				30 days postoperative complications (NA = 72)			
				No	265 (83.6)	52 (16.4)	0.167
				Yes	0 (0.0)	1 (100.0)	

Significant values are in bold.

### Conversion rate

The overall conversion rates were 0.81% (16 patients) in the renal group and 3.42% (14 patients) in the adrenal group; in patients who suffered intraoperative complications, the conversion rates were 13.01% in the renal group and 48.15% in the adrenal group.

### Short-term complications

The overall short-term complication rate was 13.45% (55) in the adrenal group and 16.44% (323) in the renal group (Table 1).

### Factors predicting intraoperative complications

The results of the univariate analysis are shown in Tables 2 and 3. In the adrenal group, the multivariate analysis revealed that the presence of invasive features was a borderline predictive factor for intraoperative complications (OR 3.57, *p* = 0.0708). In the renal group, sex, BMI, Charlson Comorbidity Index (CCI) score, surgeon experience, and cTNM were not significant. According to our multivariate analysis, the presence of malignant disease and the use of a retroperitoneal approach were protective factors against intraoperative complications (OR 0.400, *p* = 0.012 and OR 0.218, *p* = 0.001, respectively). With regard to patients who underwent partial nephrectomy, according to both univariate and multivariate analyses, a higher RENAL score was associated with a higher incidence of intraoperative complications (OR 1.279, *p* < 0.001).

**Table 3:** Univariate analysis for factors associated with intraoperative and short term post-operative complications in the renal surgery group (*n* = 1965).

	Intraoperative complications		*p*-value		Short term complications		*p*-value
	0 (*n* = 1842)	1 (*n* = 123)			0 (n = 1642)	1 (n = 323)	
Age (NA = 11)	59.91 ± 14.72	62.91 ± 12.99	0.065	Age (NA = 11)	57.33 ± 15.33	64.27 ± 12.09	**< 0.001**
Gender (NA = 8)				Gender (NA = 8)			
Male	816 (91.4)	77 (8.6)	0.369	Male	733 (78.1)	205 (21.9)	**< 0.001**
Female	579 (92.8)	45 (7.2)		Female	665 (85.0)	117 (15.0)	
BMI (NA = 523)	26.76 ± 4.57	26.50 ± 3.81	0.536	BMI (NA = 523)	27.26 ± 4.96	26.01 ± 4.20	**< 0.001**
Diabetes (NA = 713)				Diabetes (NA = 713)			
No	930 (91.1)	91 (8.9)	**0.012**	No	722 (74.1)	253 (25.9)	0.415
Type 1	53 (100.0)	0 (0.0)		Type 1	31 (81.6)	7 (18.4)	
Type 2	148 (88.1)	20 (11.9)		Type 2	121 (71.2)	49 (28.8)	
ASA (NA = 8)	2.00 (2.00–3.00)	2.00 (2.00–3.00)	0.683	ASA (NA = 8)	2.00 (1.00–3.00)	2.00 (2.00–3.00)	**< 0.001**
CCI (NA = 897)	3.00 (2.00–4.00)	3.00 (2.00–4.00)	0.149	CCI (NA = 897)	3.00 (2.00–4.00)	4.00 (2.00–5.00)	**< 0.001**
Increased preoperative bleeding risk (NA = 173)				Increased preoperative bleeding risk (NA = 173)			
No	889 (91.8)	79 (8.2)	0.999	No	999 (87.2)	147 (12.8)	**< 0.001**
Yes	493 (91.8)	44 (8.2)		Yes	398 (71.7)	157 (28.3)	
Pathology (NA = 11)				Pathology (NA = 11)			
Malignant	449 (95.3)	22 (4.7)	**0.001**	Malignant	547 (92.4)	45 (7.6)	**< 0.001**
Benign	943 (90.3)	101 (9.7)		Benign	852 (75.5)	277 (24.5)	
Padua risk category				Padua risk category			
1	185 (95.4)	9 (4.6)	**0.017**	1	164 (81.6)	37 (18.4)	**0.019**
2	131 (94.9)	7 (5.1)		2	101 (69.2)	45 (30.8)	
3	49 (84.5)	9 (15.5)		3	42 (71.2)	17 (28.8)	
Renal score	6.00 (5.00–7.00)	7.00 (6.00–8.00)	**0.018**	Renal score	6.00 (5.00–7.00)	6.00 (5.00–7.00)	0.769
cTNM	2.00 (1.00–4.00)	2.00 (1.00–4.00)	0.516	cTNM	2.00 (1.00–3.00)	3.00 (1.00–4.00)	**< 0.001**
Operative time (NA = 55)	170.00 (120.00–210.00)	210.00 (180.00–255.00)	< 0.001	Intraoperative complications (NA = 444)			
Surgeon experience (NA = 7)				No	1044 (79.8)	264 (20.2)	**< 0.001**
Senior	1265 (92.0)	110 (8.0)	0.787	Yes	74 (62.7)	44 (37.3)	
Young	131 (91.0)	13 (9.0)		Operative time (NA = 55)	150.00 (110.00–194.75)	190.00 (150.00–255.00)	**< 0.001**
				Surgeon (NA = 7)			
				Senior	1299 (82.3)	279 (17.7)	**< 0.001**
				Young	101 (69.7)	44 (30.3)	
Type of intervention (NA = 7)				Type of intervention (NA = 7)			
Partial nephrectomy	582 (89.3)	70 (10.7)	**< 0.001**	Partial nephrectomy	538 (80.4)	131 (19.6)	**< 0.001**
Radical nephrectomy	543 (92.7)	43 (7.3)		Radical nephrectomy	640 (82.1)	140 (17.9)	
Nephroureterectomy	111 (93.3)	8 (6.7)		Nephroureterectomy	61 (54.5)	51 (45.5)	
Pyeloplasty	127 (99.2)	1 (0.8)		Pyeloplasty	127 (99.2)	1 (0.8)	
Pielolythotomy	33 (97.1)	1 (2.9)		Pielolythotomy	34 (100.0)	0 (0.0)	
Surgical approach (NA = 33)				Surgical approach (NA = 33)			
Transperitoneal	1180 (91.6)	108 (8.4)	**0.015**	Transperitoneal	1252 (83.9)	240 (16.1)	**< 0.001**
Retroperitoneal	191 (96.0)	8 (4.0)		Retroperitoneal	139 (70.6)	58 (29.4)	
Hand assisted	6 (75.0)	2 (25.0)		Hand assisted	4 (50.0)	4 (50.0)	
Drainage (NA = 249)				Drainage (NA = 249)			
No	149 (100.0)	0 (0.0)	**< 0.001**	No	138 (95.2)	7 (4.8)	**< 0.001**
Yes	1016 (90.1)	112 (9.9)		Yes	1113 (79.8)	282 (20.2)	
Intraoperative blood transfusion (NA = 672)				Intraoperative blood transfusion (NA = 672)			
0	823 (96.3)	32 (3.7)	< 0.001	No	1004 (87.7)	141 (12.3)	**0.001**
1	6 (33.3)	12 (66.7)		Yes	10 (55.6)	8 (44.4)	
Short term complications (NA = 242)							
No	1044 (93.4)	74 (6.6)	< 0.001				
Yes	264 (85.7)	44 (14.3)					
30 days postoperative complications (NA = 200)				30 days postoperative complications (NA = 200)			
No	1225 (93.0)	92 (7.0)	< 0.001	No	1358 (87.3)	197 (12.7)	**< 0.001**
Yes	132 (82.0)	29 (18.0)		Yes	41 (26.8)	112 (73.2)	
Postoperative blood transfusion (NA = 504)				Postoperative blood transfusion (NA = 504)			
No	1045 (96.0)	43 (4.0)	< 0.001	No	1157 (88.1)	157 (11.9)	**< 0.001**
Yes	69 (84.1)	13 (15.9)		Yes	14 (15.9)	74 (84.1)	

Significant values are in bold.

### Predictive factors of short-term complications

The results of the univariate analysis are shown in Tables 3 and 4. According to the multivariate analysis, female sex was a significant protective factor against short-term postoperative complications (OR 0.458, 95% CI 0.235–0.893), and a higher CCI score was a risk factor for short-term postoperative complications (OR 1.493; 95% CI 1.263–1.766). In the renal group, the multivariate analysis revealed that a higher CCI score (OR 1.152, 95% CI 1.064–1.247), hand-assisted approach (OR 4.621, 95% CI 1.130, 18.899, *p* = 0.033), and nephrouretectomy (OR 2.109, 95% CI 1.319, 3.372, *p* = 0.002) were significant predictive factors of short-term postoperative complications, with radical nephrectomy being a significant protective factor (OR 0.697, 0.499, 0.973, *p* = 0.034). According to the univariate analysis of patients who underwent renal surgery, a higher ASA score was associated with a higher rate of postoperative blood transfusion (*p* < 0.001), but there were no differences in the rate of intraoperative blood transfusion.

**Table 4:** Multivariate analysis.

Adrenal surgery		
	OR for intraoperative complications (95% IC)	*p*-value
Presence of invasive features	3.572 (0.900–14.210)	0.06

	OR for short term complications (95% IC)	*p*-value
Higher Charlson’s comorbidity index	1.493 (1.263–1.766)	< 0.001
Female sex	0.458 (0.235–0.893)	0.022

	OR for conversion (95% IC)	*p*-value
`Age at intervention	0.953 (0.908–1.000)	0.051
Young Surgeon	5.146 (1.072–24.694)	0.041
`Higher operative time	1.005 (1.000–1.011)	0.048
Presence of invasive features	14.033 (2.610–75.549)	0.002

Renal Surgery		
	OR for intraoperative complications (95% IC)	*p*-value
Malignant disease	0.400 (0.176–0.909)	0.013
Retroperitoneal approach	0.218 (0.085–0.557)	0.001
Hand assisted approach	5.640 (0.812–39.174)	0.080
RENAL score	1.279 (1.134–1.442)	< 0.001

	OR for short term complications (95% IC)	*p*-value
Retroperitoneal approach	1.281 (0.820–2.001)	0.276
Hand assisted approach	4.621 (1.130–18.899)	0.033
Radical Nephrectomy	0.697 (0.499–0.973)	0.034
Nephroureterectomy	2.109 (1.319–3.372)	0.002
Pyeloplasty	0.257 (0.032–2.023)	0.197
Charlson’s comorbidity index	1.152 (1.064–1.247)	< 0.001

	OR for conversion (95% IC)	*p*-value
Young surgeon	4.277 (0.991–18.461)	0.051
Higher operative time	1.012 (1.005–1.019)	< 0.001
Higher Charlson’s comorbidity index	1.459 (1.061–2.004)	0.020

### Factors predictive of conversion

According to the univariate analysis of patients who underwent adrenal surgery, the mass dimension was not significantly associated with conversion; however, the presence of invasive features and low surgeon experience were significantly associated with a higher conversion rate (*p* = 0.002 and *p* = 0.019, respectively). According to our multivariate analysis (Table 4), low surgeon experience (OR 5.146, 95% CI 1.072, 24.694; *p* = 0.041), a longer operation time (OR 1.005, 95% CI 1.001, 1.011; *p* = 0.048), and the presence of invasive features (OR 14.033, 95% CI 2.606, 75.549; *p* = 0.002) were significant predictive factors of conversion. In the renal group, the PADUA and RENAL scores were not associated with conversion, whereas a higher cTNM and lower surgeon experience were significantly associated with higher conversion rates (*p* = 0.001 and *p* = 0.026, respectively). According to our multivariate analysis (Table 4), low surgeon experience was a borderline risk factor for conversion (OR 4.277, *p* = 0.051), and a longer operation time (OR 1.012, IC 1.001, 1.019, *p* < 0.001) and a higher CCI score (OR 1.459, IC 1.061, 2.004, *p* value 0.020) were significant predictive factors for conversion.

## Discussion

Since its introduction at the beginning of the twentieth century, laparoscopic surgery of the retroperitoneal organs has gained interest and enthusiasm. However, in addition to its known advantages, laparoscopy is not free from the risk of intraoperative complications, and their incidence may be underestimated outside major referral centres. Overall, in this study, the rate of intraoperative complications was 6.75% in the adrenal surgery group, with the most common complication reported being parenchymatous viscous complications. Apparently, this rate is higher than that reported in a large prospective multicentre study by Bergamini et al., who reported an overall perioperative complication rate of 7.9%, and a intraoperative accident rate of 3.6%. However, considering the rate of intraoperative complications reported in referral and nonreferral centres (2% and 8.2%, respectively), it appears that in nonreferral centres, the rate is similar (6.75% vs. 8.2%), thus highlighting not only the inhomogeneity but also the robustness of the data from this study, which represents the clinical reality worldwide. Furthermore, the rate of complications reported during and immediately after laparoscopic adrenalectomy is inhomogeneous; some authors have reported a rate of 4.9%^17^, whereas others^18^ have reported a rate of intraoperative complications of 33.3%. This discrepancy reflects the heterogeneity of studies that included adrenalectomy for pheocromocytoma, for which higher rates of perioperative complications are reported, mostly hypertensive crisis, and surgery for other indications. Surprisingly, this study showed that the most common intraoperative complication was parenchymatous viscous complications, with vascular complications occurring at a lower rate. These results contrast with the results of a review by Strebel et al.^15^, who reported that the most common complication of laparoscopic adrenal surgery was vascular injury^5^. This finding was probably due to a greater focus on potentially serious vascular complications than on parenchymatous visceral injuries. The results obtained in this study, on the other hand, showed that in routine surgical practice, the most frequent injuries were to parenchymatous organs, which often have no counterpart in terms of worsening postoperative outcomes and are therefore generally not considered or misrecognized in the literature. According to a multi-institutional retrospective study conducted in 2011, known risk factors for the occurrence of intraoperative complications in laparoscopic adrenal surgery include low surgeon experience, pheochromocytoma, age, BMI, and mass dimensions^19^. Nevertheless, whether mass size is a risk factor for poor surgical outcomes of laparoscopic surgery is unclear. Bergamini et al.^17^ and Shen et al.^20^ reported that a larger adrenal mass influences laparoscopic outcomes, whereas other authors did not^21–23^. The results of this study suggest that the presence of imaging features indicating local invasion (infiltration of the surrounding structures, venous invasion, and absence of an adipose cleavage plane), rather than the mass dimension^20^, could be a predictive factor of intraoperative complications and conversion, thus underlining the importance of preoperative radiological assessment to accurately clarify the morphological characteristics of adrenal masses for planning surgery. A second pivotal theme regards the role of surgical experience as a protective factor against complications, as some authors have reported that surgical volume is a predictor of better outcomes in patients undergoing adrenalectomy^24^. In this study, no significant relationship was found between surgical experience and complications. However, in line with the findings of other authors^1,15,25,26^, surgical experience was a predictive factor for conversion (*p* = 0.019). In this multicentre study, the overall short-term complication rate in patients who underwent adrenal surgery was 13.45%. This rate is slightly higher than that reported in the literature^17^. Notably, the rate of short-term complications was significantly higher than the rate of intraoperative complications, and most published studies have focused on postoperative outcomes to establish the safety of laparoscopic surgery; however, these results emphasize that postoperative outcomes alone could mask the real advantages and disadvantages of the laparoscopic approach. In the renal group, the rate of intraoperative complications was 8.09%, and the most common complications reported were hollow viscous and vascular complications. Overall, the rate of complications reported was similar to that previously reported in monocentric retrospective studies, suggesting that this could represent the average complication rate for renal laparoscopic surgery. Apparently, the proportion of hollow viscus complications was greater than that reported in other single-centre studies^11,27^. However, the majority of hollow viscous complications involved upper urinary tract structures (the calyx and renal pelvis), whereas bowel injuries were reported in only ten patients, reflecting a low rate of gastrointestinal injury. Surprisingly, although most nephrectomies were performed by urologists, the most common hollow viscous involved during partial nephrectomy was the calyx and renal pelvis. In this study, the rate of vascular complications was similar to the rate of hollow viscous complications (39.3%). Of these, 24 were classified as minor vascular complications and were managed through sutures or open conversion, and 26 were classified as major vascular complications and were managed mainly through open conversion. In contrast to the findings of other studies^28^, preoperative factors such as age and ASA score did not result in a higher complication rate. In contrast to the findings reported by other authors^29^, the rates of vascular and hollow viscous complications were comparable for patients treated via the transperitoneal approach. In this study, the retroperitoneal approach was found to be a protective factor against intraoperative complications, in line with the literature. It is conceivable that the retroperitoneal approach could be safer for patients who previously underwent abdominal surgery because it avoids the need for adhesion lysis. Another explanation for the protective role could be that surgeons who use the retroperitoneal approach have more experienced than those who use only transperitoneal access or who treat less difficult cases. In patients who underwent partial nephrectomy, a higher RENAL score was associated with a higher incidence of intraoperative complications (OR 1.279, *p* < 0.001), indicating the difficulty of these surgical procedures. With regard to the pathological characteristics of the disease, in the univariate analysis, malignant disease was associated with a higher incidence of intraoperative complications, whereas in the multivariate analysis, it was a protective factor. It is conceivable that the results of the univariate analysis did not take into account the role of confounding variables, and the statistical analysis showed that patients with malignant disease who experienced intraoperative complications had a significantly higher RENAL score than patients with benign disease who experienced intraoperative complications. Moreover, the percentage of procedures performed by young surgeons for patients with benign disease who experienced intraoperative complications was not significantly higher than the percentage of procedures performed by senior surgeons for patients with malignant diseases; therefore, neither surgeon experience could explain this result. In contrast, it seems that the type of surgery performed could have influenced the outcome in patients with benign disease since the proportion of patients who underwent partial nephrectomy was significantly higher in patients with benign disease who experienced intraoperative complications than in patients with malignant disease who did not experience intraoperative complications (*p* = 0.005). With regard to postoperative complications, preoperative factors, depending upon patient comorbidities, such as a higher CCI score (OR 1.152, 95% CI 1.064–1.247), hand-assisted approach (OR 4.621, 95% CI 1.130, 18.899, *p* = 0.033) and nephroureterectomy (OR 2.109, 95% CI 1.319, 3.372, *p* = 0.002) were significant predictive factors of short-term postoperative complications, whereas radical nephrectomy was a protective factor for short-term postoperative complications (OR 0.697, 95% CI 0.499, 0.973, *p* = 0.034). These results are not surprising considering the greater technical difficulties associated with nephroureterectomy than with radical nephrectomy. With regard to the hand-assisted laparoscopic approach, we know that this approach is generally used by surgeons with the aim of providing better control of potential vascular complications (i.e., massive bleeding from the main vessels) rather than acting as a “retractor” into the abdominal cavity. Furthermore, compared with pure laparoscopy, the hand-assisted technique might provide faster organ removal (i.e., kidney extraction) once the procedure has been finalized. Despite the theoretical advantages of the hand-assisted approach, it is no longer adopted by more skilled laparoscopic surgeons who prefer pure conventional laparoscopy, even for the most challenging cases. Indeed, hand-assisted surgery is mostly performed by colleagues with less experience as a sort of “safer approach”; however, limited surgical experience can often result in more intra- and postoperative complications (an increased overall complication rate). On the other hand, in this study, surgeon experience did not affect the results for the following reasons. In all the centres included in our analysis (i.e., academic hospitals), when junior colleagues and/or less skilled surgeons performed the procedures (especially those that were technically demanding and difficult), they worked alongside more experienced surgeons to avoid and, if needed, properly manage complications. As a result, we minimized any risk for the patients, and did not therefore significantly affect the outcomes during the process of mastering the surgical learning curve. Finally, with regard to factors influencing the need for perioperative blood transfusions, the results of this study suggest that the postoperative blood transfusion rates are not related to the type of surgical procedure but instead depend on patient preoperative risks and comorbidities. This study has several limitations. First, the same surgical region (retroperitoneal space) was used for each of the different surgeries. Second, a subgroup analysis for benign and malignant diseases was not performed, even though malignant masses are more challenging to access than benign masses. Finally, it could be argued that the study being a retrospective multicentre study lowered the weight of the results. However, the retrospective design is not a limitation, as it avoids distortion of clinical reality and surgeons’ reticence in reporting intraoperative complications. Despite these limitations, this study provides valuable insights into the possible disadvantages of laparoscopic surgery for tumours involving the adrenal gland and kidney. The results of this study can be translated to clinical practice, even in centres that not considered high-volume centres, and can be used to identify tumour-related factors that can predict intraoperative complications.

## Conclusion

The results of this multicentre international study showed overall intraoperative complication rates of 6.75% for laparoscopic adrenal surgery and 8.09% for laparoscopic renal surgery. The multivariate analysis revealed that radiological preoperative factors, such as invasive features for patients undergoing adrenalectomy and the RENAL score for those undergoing nephrectomy, are predictive factors of intraoperative complications. In contrast to existing data, surgeon experience was not associated with a reduction in the incidence of perioperative complications.

## Data Availability

The datasets used and/or analysed during the current study are available from the corresponding author on reasonable request.
